# Polyploidy-induced senescence: Linking development, differentiation, repair, and (possibly) cancer?

**DOI:** 10.18632/aging.206355

**Published:** 2026-02-08

**Authors:** Iman M. Al-Naggar, George A. Kuchel

**Affiliations:** 1 Department of Cell Biology, University of Connecticut School of Medicine, Farmington, CT 06030, USA; 2 Department of Surgery (Division of Urology), UConn Health, Farmington, CT 06030, USA; 3 University of Connecticut Center on Aging, Farmington, CT 06030, USA

**Keywords:** cellular senescence, cancer, polyploidization, differentiation, urothelial carcinoma, oncogene-induced senescence

Since the first description of replicative senescence triggered by telomere shortening in the 1960s, other stressors such as mitochondrial dysfunction and DNA damage were shown to induce senescence *in vitro*. *In vivo*, senescent cells show both beneficial physiological and harmful pathological roles, yet their contribution to aging and disease remain incompletely understood [1]. One understudied form of cellular senescence is polyploidy-induced senescence (PIS) which was initially observed *in vitro* after drug-induced tetraploidization [2]. We recently reported that polyploid uroepithelial cells in the mouse bladder are senescent over the lifespan, raising new questions about the physiological and pathological significance of polyploid, senescent cells [3]. These senescent uroepithelial barrier cells persisted after treatment of mice with the senolytic combination dasatinib plus quercetin (D+Q). Although preservation of this critical blood–urine barrier during senolytic treatment may be advantageous—since extensive disruption could lead to cystitis and severe bladder symptoms—it remains unclear whether these cells are intrinsically resistant to D+Q or whether limited drug penetration, due to the bladder’s relatively low perfusion, prevented adequate exposure. This latter issue is a common obstacle to drug delivery to the bladder.

Polyploidization—whole genome duplication arising from cell fusion, failed cytokinesis, or endoreplication—confers stress resistance, enabling survival in harsh environments such as the toxic and metabolically demanding liver niche [4–6]. It also allows specialized cells such as pancreatic β-cells and mammary epithelial cells to produce large amounts of protein. Polyploid cells undergo genome reorganization, epigenetic remodeling, and activation of novel DNA repair pathways that allow them to resist oxidative stress, inflammation, and accumulating DNA mutations [5]. They also acquire stem-like gene expression by expressing stemness transcription factors such as NANOG (Homeobox Transcription Factor Nanog), OCT4 (octamer-binding transcription factor 4), and SOX2 (SRY-Box Transcription Factor 2), enabling repair and regeneration, especially in the absence of stem cells. While polyploidy is common in tumors and can occur in response to some cancer drugs (where it can result in aneuploidy, drug resistance, and metastasis) [6], normal, physiological polyploidization is developmentally programmed in many cell types, including cardiomyocytes, hepatocytes, megakaryocytes, skin, pancreatic, mammary, brain, renal, and uroepithelial cells. Thus, polyploidization can act as both a homeostatic adaptation and a potential precursor to malignancy.

Our recent study described the first example of potentially beneficial, lifelong, PIS *in vivo* [3]. Superficial mouse bladder umbrella cells, located at the urine interface, exhibit senescence markers from 2–26 months of age, suggesting a role in barrier formation, tissue structure, repair, and regeneration. These cells express high levels of Cyclin D1, a cell cycle regulator previously shown to drive premature PIS, like oncogene-induced or developmental senescence [7, 8].

Dysregulation of Cyclin D1 can be oncogenic, which is traditionally attributed to its interaction with Cyclin Dependent Kinases 4 and 6 (CDK4/6) and its role in cell cycle progression. However, recent studies have revealed non-canonical roles of Cyclin D1 via its intrinsically disordered domain, independently of its CDK4/6 interacting site. These include roles in transcriptional regulation, DNA damage response initiation (via recruitment to histone H2B containing the phosphorylated Serine 14 which co-localizes with γH2AX in DNA damage foci), and genome stability. These latter functions of Cyclin D1 may account for its enrichment in polyploid, senescent umbrella cells [9]. This functional duality may underlie both the inconsistent efficacy of Cyclin D1–targeted therapies and its contradictory prognostic value across cancers. Our findings, together with emerging evidence that Cyclin D1 contributes to induction of chromosomal instability (CIN), argue for a re-evaluation of the mechanism of its causal role in cancer initiation. Clarifying Cyclin D1’s roles in physiological polyploidy and senescence may ultimately refine our understanding of the molecular mechanisms governing cancer initiation, progression, and malignancy in the bladder and other tissues.

Polyploidy-induced senescence, PIS, depends on intact tumor suppressor machinery. Panopoulos et al. [2] showed that p16 (CDKN2A, Cyclin-dependent kinase inhibitor 2A) is required for PIS; in its absence, such as in many transformed cell lines, or when the Retinoblastoma or p53/p21 pathways are disrupted, polyploid cells fail to arrest. Moreover, frequent loss of chromosome 9p21, which harbors both tumor suppressors p16 and p14ARF, is a clinical marker in bladder cancer and multiple other cancers. We now hypothesize that some bladder cancers, 90% of which are of urothelial origin, may arise from polyploid umbrella cells that, through loss of senescence enforcers and tumor suppressors such as p16, escaped PIS.

The idea that cancers can arise from cells escaping senescence is well established [10], but our observations link this specifically to polyploidization. This has important implications in the context of therapy-induced senescence (TIS). Many cancer treatments trigger senescence through replication stress and polyploidization [11]. While initially growth-arrested, these polyploid cancer cells can adapt, escape, and drive relapse, and caution has been proposed with the use of drugs that cause cancer cells to become polyploid and resistant to treatment [6]. By contrast, naturally occurring polyploid senescent cells, such as bladder umbrella cells, appear to serve important biological functions—though they too may destabilize under chronic stress. For example, smoking, the leading risk factor for bladder cancer, introduces urinary toxins such as acrolein that increase oxidative stress and DNA damage. Such insults may erode the PIS arrest in umbrella cells, enabling their re-entry into the cell cycle, mutagenesis, and malignant transformation.

Not all polyploid cells are senescent, and their relationship is context dependent. Hepatocytes, for example, can be both polyploid and senescent, but polyploid hepatocytes also undergo senescence reversal and ploidy reduction divisions under stress, re-entering the cell cycle and contributing to carcinogenesis [12]. We propose that PIS acts as a developmental timer: replication stress from endoreplication activates the DNA damage response, linking proliferation to differentiation during development, regeneration and repair. In this model, senescence is not merely a stress response but a programmed cellular fate that enforces terminal differentiation, contributes to organ structure, and preserves tissue architecture.

Until recently, much of what we knew about senescent cells came from cultured cells exposed to stressors *in vitro* (e.g., DNA-damaging drugs such as doxorubicin, radiation, or increased oxidative stress via hydrogen peroxide). Nuclei of cells treated in this manner typically increased in size, a strong marker of polyploidization, but most studies did not measure ploidy and senescence simultaneously. Thus, it is unknown what proportion of senescent cells arose in response to polyploidization in these early cell culture models. Carefully untangling polyploidization from senescence, their effect on each other, and the contribution of their inherent characteristics or changes that disrupt each of them in normal tissues with aging or other exposures (e.g., chemotherapeutics, smoking), may have important implications for our understanding of tumorigenesis, especially vis-à-vis the molecular mechanisms driving cancer initiation.

Disentangling the overlap between polyploidy and senescence, however, remains a major challenge. Large-scale efforts such as the National Institutes of Health Common Fund’s Cellular Senescence Network Program (SenNet) are beginning to map senescent cells across human tissues. To fully capture the biology of PIS, these initiatives must also assess ploidy and avoid relying on methods such as conventional single nuclear RNA sequencing which may exclude polyploid or multinucleated cells from analyses. Such methodological refinements are essential for clarifying how PIS contributes to tissue structure, maintenance, dysfunction, and transformation.

## CONCLUSIONS

**Figure 1:**
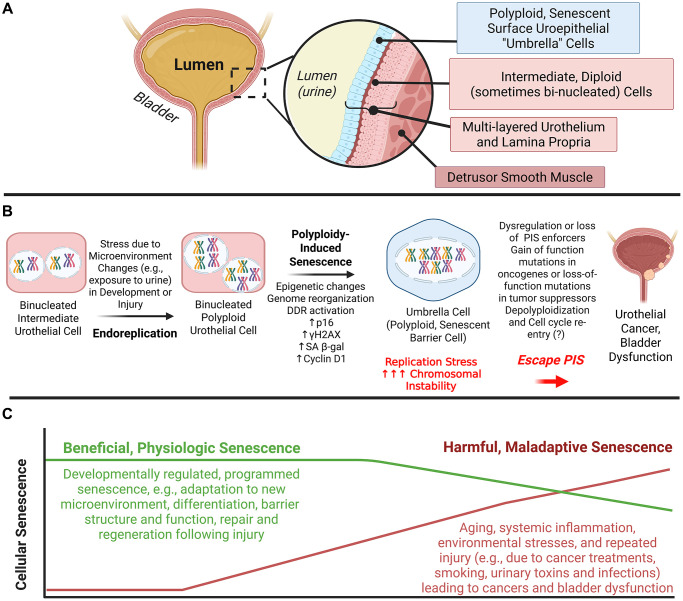
**Polyploidy-induced senescence and its potential role in bladder dysfunction and carcinogenesis.** (**A**) Polyploid bladder superficial uroepithelial cells (“Umbrella Cells”) at the bladder-urine interface exhibit markers of cellular senescence throughout the lifespan in mice. (**B**) Programmed somatic polyploidy is typically associated with terminal tissue differentiation. Polyploid umbrella cells result from endoreplication during development or repair and undergo polyploidy-induced senescence (PIS). We hypothesize that replication stress in these cells can result in increased chromosomal instability, resulting in the loss of senescence enforcers (e.g., p16). As a result, some polyploid cells can “depolyploidize” and reenter the cell cycle. This error-prone “return to division” process increases the likelihood of producing aneuploid cells, and although most progeny of depolyploidized cells die, a subset survives and continues proliferating, often acquiring genetic and epigenetic alterations that promote tumorigenesis in these highly resilient polyploid cells which can result in a growth advantage and aggressive carcinomas. (**C**) Developmentally regulated PIS exists in multiple organs and plays important physiological roles, allowing cells to survive their harsh microenvironments and continue to carry out their function (e.g., maintaining the urine-blood barrier in the bladder). However, mechanisms important for regulation of this beneficial senescence may become dysregulated, giving rise to harmful senescence and cancers. Harmful senescent cells can also arise from other biological mechanisms (e.g., DNA damage, mitochondrial dysfunction, inflammation, oxidative stress) common in aging and other conditions. PIS: Polyploidy-Induced Senescence, DDR: DNA Damage Response, SA β-gal: senescence associated β-galactosidase, γH2AX: phosphorylated histone variant H2AX. This figure was created using Biorender.

Polyploidization and senescence may be interrelated stress responses, yet they have been studied mostly in isolation. Polyploid cells exist in many organs prone to cancer, but their senescence status is not well defined. Polyploidy confers survival advantages—stress resistance, stemness, immune and apoptosis evasion—while senescence enforces arrest, and together, they may function as a developmental differentiation timer for establishing and maintaining the bladder uroepithelial layer’s structure and function. However, as polyploid senescent cells accumulate mutations (e.g., gain-of-function mutations in oncogenes, and/or loss-of-function mutations in tumor suppressors) or lose senescence regulators such as p16 (through epigenetic silencing or deletion), they may escape arrest and become invasive cancers (Figure 1). Our work highlights the need to study polyploidy and senescence in concert to understand their roles in aging, cancer, and therapeutic resistance.
